# Lifshitz transition from valence fluctuations in YbAl_3_

**DOI:** 10.1038/s41467-017-00946-1

**Published:** 2017-10-11

**Authors:** Shouvik Chatterjee, Jacob P. Ruf, Haofei I. Wei, Kenneth D. Finkelstein, Darrell G. Schlom, Kyle M. Shen

**Affiliations:** 1 Laboratory of Atomic and Solid State Physics, Department of Physics, Cornell University, Ithaca, NY 14853 USA; 2 Cornell High Energy Synchrotron Source, Wilson Laboratory, Cornell University, Ithaca, NY 14853 USA; 3 Department of Materials Science and Engineering, Cornell University, Ithaca, NY 14853 USA; 4 Kavli Institute at Cornell for Nanoscale Science, Ithaca, NY 14853 USA; 5 Present Address: Department of Electrical & Computer Engineering, University of California, Santa Barbara, CA 93106 USA

## Abstract

In mixed-valent Kondo lattice systems, such as YbAl_3_, interactions between localized and delocalized electrons can lead to fluctuations between two different valence configurations with changing temperature or pressure. The impact of this change on the momentum-space electronic structure is essential for understanding their emergent properties, but has remained enigmatic. Here, by employing a combination of molecular beam epitaxy and in situ angle-resolved photoemission spectroscopy we show that valence fluctuations can lead to dramatic changes in the Fermi surface topology, even resulting in a Lifshitz transition. As the temperature is lowered, a small electron pocket in YbAl_3_ becomes completely unoccupied while the low-energy ytterbium (Yb) 4*f* states become increasingly itinerant, acquiring additional spectral weight, longer lifetimes, and well-defined dispersions. Our work presents a unified picture of how local valence fluctuations connect to momentum-space concepts such as band filling and Fermi surface topology in mixed valence systems.

## Abstract (web summary)

How the electronic structure of a mixed-valence system changes with respect to local chemical environment remains elusive. Here, Chatterjee et al. show that valence fluctuations of YbAl_3_ can lead to dramatic changes in the Fermi surface topology in reciprocal space.

## Introduction

Kondo lattice systems host a wide variety of quantum states such as antiferromagnetism^1^, heavy Fermi liquids^2^, hidden order^3^, and unconventional superconductivity^4^, which can often be controlled by modest perturbations using magnetic field or pressure, thereby providing access to quantum phase transitions^5–7^. These states generally emerge from a complex many-body state that is formed by enhanced Kondo coupling between the local rare-earth moments and the band-like conduction electrons at low temperatures. In mixed valence systems^8–10^, this coupling also results in a change of the rare-earth valence, which can be determined by core-level spectroscopies that probe the local chemical environment (*r*-space)^11–13^, but the implications for the momentum-space (*k*-space) electronic structure remain poorly understood. To gain insight into the emergent properties of these systems, it is crucial to understand how delocalized carriers and the low-energy momentum-space electronic structure emerge from these local interactions.

Here, we choose YbAl_3_ as a simple prototypical mixed valence system with two nearly degenerate ytterbium (Yb) valence configurations, Yb^2+^ (4*f* ^14^) and Yb^3+^ (4*f* ^13^). The average Yb valence, *ν*_f_, decreases with temperature, changing by ∼Δ*ν*_f_ = −0.05 from 300 K to below *T* ^∗^ ≈ 34–40 K^11, 12, 14–16^, when it becomes a heavy Fermi liquid, attributed to the enhanced Kondo screening at low temperatures^17^. We selected YbAl_3_ due to its relatively large change in valence as well as its large energy scales, with a reported single ion Kondo temperature *T*_K_ ≈ 670 K^17, 18^, which should make these changes observable in momentum space. The lack of a well-defined, pristine surface in cleaved YbAl_3_ single crystals^19^, however, has previously prevented momentum-resolved measurements of its electronic structure. We have circumvented this problem by synthesizing epitaxial thin films of YbAl_3_ and its conventional metal analog LuAl_3_ by molecular beam epitaxy (MBE)^20^ and have combined it with in situ angle-resolved photoemission spectroscopy (ARPES) to directly measure their electronic structure as a function of temperature. Our measurements reveal a strong temperature-dependent change in both the real and momentum-space electronic structure of YbAl_3_. The local Yb valence decreases as the temperature is lowered, accompanied by a large shift in the chemical potential which leads to a Lifshitz transition of a small electron pocket at Γ, along with the emergence of renormalized heavy quasiparticles near the Fermi energy (*E*_F_). We establish a direct one-to-one correspondence between these observed changes, which we believe to be generic to all mixed valence systems.

## Results

### Synthesis and electronic structure

**Fig. 1:**
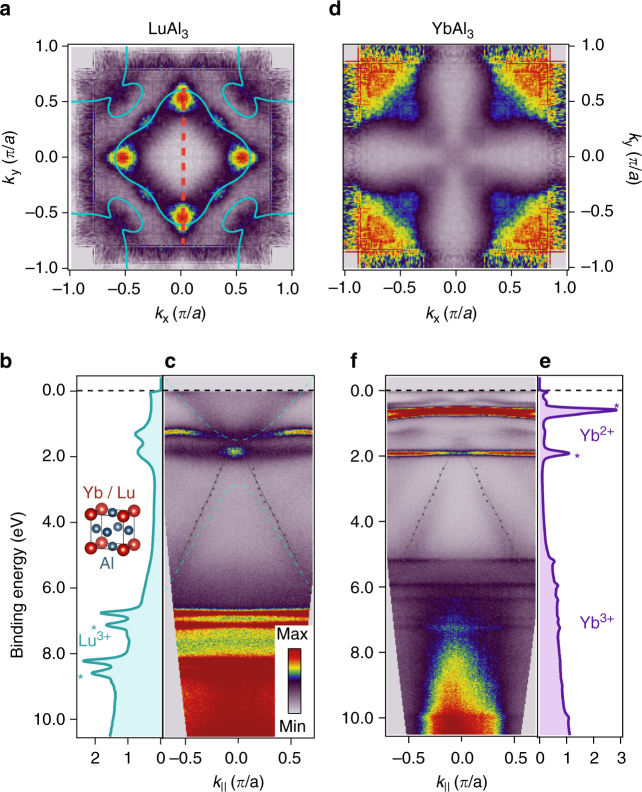
Electronic structure and Fermi surfaces of YbAl_3_ and LuAl_3_. Fermi surface maps and energy distribution curves (EDCs) and *E* vs. *k* dispersion for **a**–**c**, LuAl_3_ and **d**–**f**, YbAl_3_, all measured with *hν* = 21.2 eV at 21 K. Experimental Fermi surfaces of **a**, LuAl_3_ and **d**, YbAl_3_. DFT calculations (green lines) of the Fermi surface topology at *k*_*z*_ = 0 for LuAl_3_ with *U* = 0 are overlaid in **a**. Momentum-integrated EDCs of **b**, LuAl_3_ and **e**, YbAl_3_, with surface core levels marked as asterisks. *E* vs. *k* dispersions for **c**, LuAl_3_ and **f**, YbAl_3_, together with DFT calculations of the band structure in LuAl_3_ (green) shown in **c**. The similarity between the dispersion of the light band between 2 and 6 eV in YbAl_3_ and LuAl_3_ suggest that both compounds have similar inner potentials, and that both measurements are at *k*_z_ = 0 ± 0.1*π*/*a*

Both YbAl_3_ and LuAl_3_ crystallize in a cubic *Pm* $\bar 3$ *m* structure where Yb or Lu atoms occupy the vertices of the unit cell while Al atoms occupy the face centers, as illustrated in the inset of Fig. 1b. LuAl_3_ has fully occupied 4*f* orbitals with zero net moment and a lattice constant (4.19 Å) closely matched to YbAl_3_ (4.20 Å). Thus, LuAl_3_ serves as an ideal reference compound to understand the light, Al-derived band-like conduction electron states, which are also common to YbAl_3_. Epitaxial thin films of both LuAl_3_ and YbAl_3_ with (001) out-of-plane orientation were synthesized by co-evaporation on MgO (001) substrates (4.21 Å) at temperatures of 200–350 °C and a chamber base pressure below 2 × 10^−9^ Torr. For all films, a 1.2 nm thick aluminum (4.05 Å) buffer layer was deposited at 500 °C, which allowed the growth of continuous, smooth films of LuAl_3_/YbAl_3_ on top. In these studies, we investigated a 30 nm thick LuAl_3_ film and a 20 nm thick YbAl_3_ film (the YbAl_3_ was synthesized on top of a 20 nm thick LuAl_3_ buffer layer on top of the Al buffer, which improved the quality of the YbAl_3_ layers). All films were sufficiently thick so that any photoemission intensity from the buffer layers or substrate and thickness-dependent finite size effects can be ignored. Additional details about the synthesis can be found in “Methods” section as well in ref. ^20^.

In Fig. 1, we show Fermi surface maps and the electronic structure from LuAl_3_ and YbAl_3_ thin films from the Fermi energy (*E*_F_) to a binding energy of 10.5 eV. For LuAl_3_, only the Lu 4*f* ^13^ final states are observed, with the *J* = 7/2 and 5/2 core levels at binding energies of 6.7 and 8.2 eV, respectively. Highly dispersive Al-derived bands can be observed in both LuAl_3_ (Fig. 1c) and YbAl_3_ (Fig. 1f), which extend from about 6 eV binding energy to near *E*_F_. By matching the experimentally determined dispersion of these bands as well the Fermi surface contours measured with both He I*α* (21.2 eV) and He II*α* (40.8 eV) photons to density functional theory (DFT) calculations, we are able to determine our out-of-plane momenta (*k*_z_) values for both YbAl_3_ and LuAl_3_. Due to the lack of strong correlations in LuAl_3_ (its 4*f* shell is entirely filled), DFT calculations should accurately describe its electronic structure and indeed we find good agreement between both the DFT-calculated band dispersions as well as Fermi surface contours to the experimentally determined dispersions and Fermi surface from ARPES, assuming an inner potential of *V*_0_ − *ϕ* = 13.66 eV (Fig. 1a, c and Supplementary Fig. 1). We observe broadly dispersive, primarily Al-derived bands in YbAl_3_ (Fig. 1f) analogous to those observed in LuAl_3_, and also found excellent correspondence between the measured electronic structure in LuAl_3_ and YbAl_3_ over the entire Brillouin zone (Supplementary Fig. 3) indicating that we are probing a similar *k*_z_ as in LuAl_3_, as one might expect given their highly similar electronic and crystal structures. Using the value of *V*_0_ − *ϕ*, we determine that for *hν* = 21.2 eV, we are probing near the zone center, Γ, *k*_z_ = 0 ± 0.1*π*/c. More details about the *k*_z_ determination can be found in Supplementary Note 1.

A two dimensional slice at *k*_z_ = Γ of the three-dimensional Fermi surface of LuAl_3_ accesses a multiply connected Fermi surface sheet consisting of electron-like pockets centered at (0, 0) and (*π*, *π*), consistent with our ARPES data, shown in Fig. 1a–c. On the other hand, in YbAl_3_, we clearly observe both the Yb 4*f* ^13^ and 4*f* ^12^ final states around 0–2 eV and 6–10.5 eV binding energy, respectively, consistent with its mixed valence character. The near-*E*_F_ electronic structure in YbAl_3_ is, however, significantly modified by a shift in its chemical potential due to the differing average Lu and Yb valence and the interaction between the broad, dispersive bands and the renormalized Yb 4*f* states. In the Fermi surface map of YbAl_3_ (Fig. 1d), large Fermi surface sheets are prominent and centered at zone edges (*π*, *π*).

### Evolution of the electron pocket at Γ

**Fig. 2:**
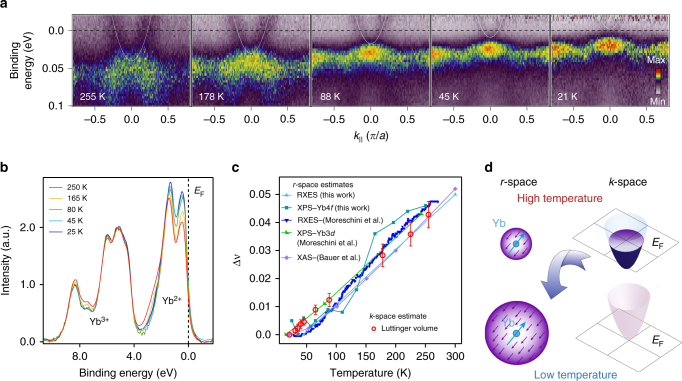
Correspondence between *r*-space and *k*-space electronic structure in YbAl_3_. **a** Evolution of the low-energy electronic structure with temperature. *E* vs. *k* dispersions are divided by the corresponding resolution-broadened Fermi-Dirac distribution to emphasize thermally occupied states above *E*_F_. White lines are guides to the eye showing evolution of the electron-like pocket centered at (0, 0, 0). **b** XPS spectra showing the temperature-dependent intensity variation of the 4*f* ^13^ and 4*f* ^12^ final states in YbAl_3_, after Shirley background subtraction^37^ and normalized by the 4*f* ^12^ final state intensity. **c** Temperature dependence of the change in Luttinger volume, estimated from the size of the electron pocket at (0, 0, 0) and of the change in Yb valence, measured by core-level spectroscopy, revealing a precise one-to-one correspondence. Error bars reflect uncertainty in the Luttinger volume estimation due to a statistical error of one standard deviation in the extracted *k*_F_ values from the fits to the Momentum distribution curves (MDCs) taken at *E*_F_. **d** Schematic illustrating the temperature-dependent relationship between *r*-space and *k*-space electronic structure in YbAl_3_

Having discussed the basic electronic structure, we now turn towards its temperature dependence in YbAl_3_. In Fig. 2a, we show a series of ARPES spectra obtained along (0, 0) to (0, *π*) at *k*_z_ ≈ Γ between 255 and 21 K, which establish a clear temperature-dependent shift of the chemical potential Δ*μ* with the 4*f*-derived states moving closer to *E*_F_ as the temperature is lowered, consistent with earlier angle-integrated measurements^14^. The most dramatic effect of Δ*μ* is on a small parabolic electron pocket centered at Γ. At 255 K, the electron pocket can be clearly observed with its band bottom at 40 ± 5 meV binding energy and a *k*_F_ of 0.20 ± 0.01*π*/*a*. As the temperature is lowered, the electron pocket is lifted in energy and becomes entirely unoccupied around 21 K. Since the pocket is centered at Γ, its lifting above *E*_F_ would then coincide with a Lifshitz transition. To within experimental resolution, the dispersion or effective mass of the electron pocket does not change apart from a rigid shift due to Δ*μ*. Furthermore, while the Yb 4*f* ^13^ final states also shifted in energy, the Yb 4*f* ^12^ states did not shift appreciably with temperature indicating that the Δ*μ* shift arises from an alteration in band filling due to the emergence of a Kondo screened many-body state.

We note that the small electron pocket at Γ is not reported in previous de Haas-van Alphen (dHvA) studies of YbAl_3_, which can be explained by the fact that the electron pocket is only occupied at higher temperatures (*T* > 20 K), whereas dHvA measurements are conducted at low temperatures (*T* ≈ 20 mK-1.5 K)^18, 21^. Nevertheless, DFT calculations suggest the presence of a quasi-spherical electron pocket at Γ, but whose size strongly depends on the binding energy of the Yb 4*f* states (Supplementary Fig. 4), and thus the value of *U* used in the calculations. Furthermore, our observation of the temperature-dependent chemical potential shift may explain the need to artificially shift the chemical potential in previous low-temperature quantum oscillation experiments of YbAl_3_ which compared their results to band structure calculations^21^. The large Fermi surface sheets centered at (*π*, *π*) (see Fig. 1d and Supplementary Fig. 2) measured by ARPES would give an oscillation frequency of ≈1.0 ± 0.2 × 10^8^ Oe, which is comparable but somewhat larger than the largest reported quantum oscillation frequency along the (100) direction, 6.51 × 10^7^ Oe. This discrepancy might be due to the fact that the quantum oscillations are measuring a closed Fermi surface contour at a different *k*_z_ than our ARPES measurements at *k*_z_ = 0, which might correspond to an open contour. We also observed another Fermi surface sheet centered at the M point (*π*, *π*) with 40.8 eV photon energy which corresponds to an oscillation frequency of 3.5 × 10^7^ ± 1 × 10^7^, roughly consistent with the *β* pocket reported in the dHvA measurements (4.55 × 10^7^ Oe).

In Fig. 2b, we show a series of angle-integrated wide energy valence band in situ x-ray photoemission spectra (XPS) showing a dramatic temperature-dependent change in relative intensity of the 4*f* ^13^ and 4*f* ^12^ final states. As the temperature is lowered, the relative intensity of the 4*f* ^13^ final states increases, while that of the 4*f* ^12^ final states decreases indicating a reduction of the effective Yb valence in YbAl_3_ at lower temperatures, which is found to be Δ*ν*_f_ ≈ 0.05 from room temperature to below ≈ 45 K, in agreement with previously reported results from bulk samples^11, 12, 14–16^.

### Relation between real-space and momentum-space electronic structure

In Fig. 2c, we make a quantitative comparison between the observed change in the temperature-dependent band filling and the estimated change in the Yb valence from core-level spectroscopy, both in our thin films and previous measurements on YbAl_3_ single crystals. The change in average Yb valence in our thin films has been estimated by resonant x-ray emission spectroscopy (RXES) and XPS, details for which can be found in “Methods” section and in Supplementary Note 2. Assuming a spherical geometry $\left( {\frac{4}{3}\pi k_{\rm{F}}^{\rm{3}}} \right)$ due to its location at *k* = (0, 0, 0) and the cubic symmetry, we plot the change in Luttinger volume of the electron pocket Δ*ν*_Lutt_, vs. the estimated change in Yb valence Δ*ν*_f_, from core-level spectroscopy. Without any adjustable parameters or scaling factors, we discover a precise, one-to-one correspondence between Δ*ν*_Lutt_ from the electron pocket and Δ*ν*_f_ as a function of temperature. This provides direct microscopic evidence that in YbAl_3_, the Kondo screening of the 4*f* moments by the conduction electrons that results in the emergence of composite heavy fermion quasiparticles leads to a Lifshitz transition of the Fermi surface, which is also reflected in the reduction of the average Yb valence, and should be generic to other mixed valence systems.

A qualitative model of the temperature-dependent changes in both real and momentum-space is presented in Fig. 2d. As the temperature is lowered, the filling of the small electron pocket is gradually reduced as those electrons are transferred into the Kondo screening cloud at the Yb site leading to the formation of renormalized Kondo screened many-body states^22^ and a reduction of the effective Yb valence as measured by XPS and RXES studies. This model would explain the direct one-to-one correspondence between the measured changes in both the Yb valence and the Luttinger volume of the electron pocket as a function of temperature.

We should note that previous studies of other mixed valence systems, such as YbRh_2_Si_2_ have not reported temperature-dependent changes in the band structure or Fermi surface topology^23^, although this may have been because of the much larger Δ*ν*_f_ in YbAl_3_ (0.05 vs. 0.01) in the accessed temperature range of the experiments, as well as its larger energy scales (*T*_K_ = 670 K vs. 25 K)^23^.

### Evolution of the Yb 4*f* states

**Fig. 3:**
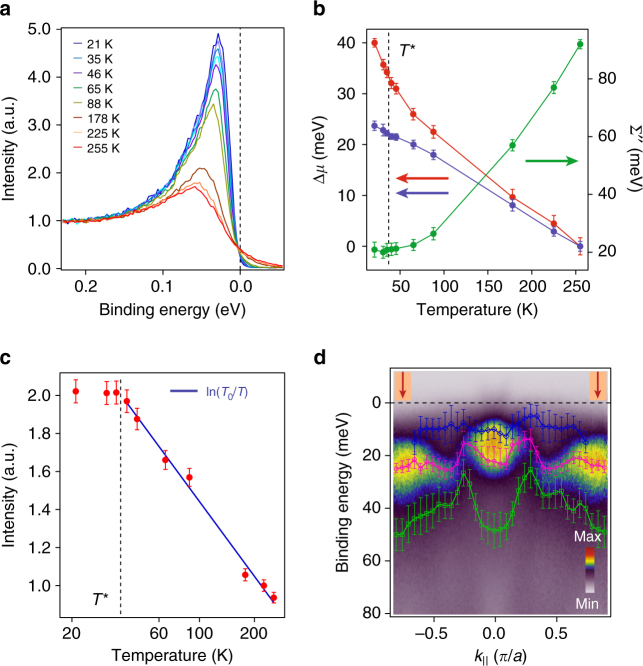
Evolution of the Yb 4*f* states and crystal field effects in YbAl_3_. **a** Evolution of the Kondo resonance peak with temperature, from integrating EDCs over *k* region highlighted as red in **d**. **b** Change in the chemical potential (Δ*μ*) and in the 4*f* quasiparticle scattering rate with temperature. Δ*μ* is estimated from the shift in binding energy of the 4*f*-derived heavy band (blue) and the band bottom of the light electron-like pocket at (0, 0) (red) relative to 255 K. Error bars represent uncertainty due to statistical error of one standard deviation from the fitting process. **c** Temperature dependence of the integrated spectral weight (0–0.2 eV) of the 4*f* states which show a ln(*T*_0_/*T*) behavior above *T* ^*^ = 37 K. Error bars represent 3% margin of error. **d** High resolution *E* vs. *k* plot along (0, 0)–(0, *π*) at 21 K showing dispersive crystal electric field (CEF) split states. Extracted dispersions of the three different CEF split states (shown in blue, pink and green) are superimposed on the image plot. In addition to statistical error of one standard deviation from the fitting process, error bars in **d**, also include variability in the fit results by holding one and/or two peak positions constant in the multi-peak fitting process

We now discuss the evolution with temperature of the 4*f*-derived heavy bands near *E*_F_. In Fig. 3a, we show representative energy distribution curves (EDCs) at different temperatures integrated over the momentum region indicated in Fig. 3d, together with extracted changes of the 4*f* binding energy, quasiparticle weight, and scattering rate as a function of temperature (Fig. 3b, c). Details of the fitting process along with extended data sets can be found in Supplementary Note 3 and Supplementary Fig. 7. We find a dramatic enhancement of the quasiparticle spectral weight of the 4*f* bands, consistent with previous measurements by Tjeng et al.^11^, coinciding with a precipitous drop in the scattering rate, which saturates around *T* ^∗^ ≈ 37 K, the estimated coherence temperature of YbAl_3_^20^ when it becomes a Fermi liquid. The enhancement of the quasiparticle spectral weight and lifetime with decreasing temperature suggests that the screening of the 4*f* moments by the conduction electrons has nearly saturated around *T* ^∗^, and that the Lifshitz transition is coincident with this dramatic change in the 4*f* spectral function. This is further highlighted by the observation of a ln(*T*_0_/*T*) ≥ 255 K scaling behavior in the integrated spectral weight, as expected from a two fluid model^22, 24–26^, until the onset of Fermi liquid behavior at *T* ^*^, when it starts to saturate. The observation of this scaling behavior up to 255 K, the highest temperature accessed in this study, suggests that the hybridization between the local 4*f* moments and the conduction electrons sets in at a relatively high temperature, even though the Fermi liquid regime exists only below *T* ^∗^, consistent with the slow crossover scenario predicted by slave boson mean field calculations^27, 28^. The saturation of the 4*f* quasiparticle lifetime at *T* ^∗^ in our ARPES measurements is also consistent with earlier transport and thermodynamic measurements, which suggested that *T* ^∗^ could be related to the formation of coherence in the 4*f* states^15, 17, 18^, which we establish spectroscopically. The shift in binding energy of the 4*f* states is smaller compared to the Δ*μ* measured from the electron-like band, with the discrepancy increasing at lower temperatures, shown in Fig. 3b, indicative of enhanced hybridization between the 4*f* states and the conduction electrons at lower temperatures that pushes the electron pocket further towards lower binding energy.

### Dispersive crystal electric field split states

In addition to the strong temperature dependence of the electronic structure, our measurements clearly show three distinct flat bands close to *E*_F_ which acquire significant dispersion at certain *k* points. Their proximity to *E*_F_, the value of their splittings (≈0–30 meV), and their narrow bandwidths are all consistent with these being crystal electric field (CEF) split states. The dispersion of the CEF states occur when the light Al-derived bands cross the flat 4*f* states near *E*_F_, lifting the degeneracy of the CEF split branches, as shown in Fig. 3d (also see Supplementary Fig. 8). The observation of three distinct bands is consistent with the bulk cubic symmetry of the Yb ions, where the Yb *J* = 7/2 manifold should split into three crystal field levels Γ_6_, Γ_7_, and Γ_8_^29^. While we cannot determine conclusively whether these states are representative of bulk vs. surface Yb atoms, the values of their splittings (0–30 meV) and bandwidths (≤ 25 meV) from ARPES and the fact that they extend from *E*_F_ to a binding energy of ≈50 meV are also consistent with reports from bulk-sensitive inelastic neutron scattering which do not observe sharp CEF excitations but rather a broad continuum (≈50 meV), since those measurements would average the dispersion of the CEF states over the entire Brillouin zone^30–32^.

## Discussion

Our work experimentally provides a unified picture of how local changes of the rare-earth valence impacts the momentum-space electronic structure in the prototypical mixed valence system, YbAl_3_. We have achieved this by combining state-of-the-art materials synthesis and advanced in situ spectroscopy, which should be readily extendable to other Kondo lattice systems or even artificial *f*-electron heterostructures. We have discovered that a Lifshitz transition of a small electron Fermi surface accompanies the change in average Yb valence, which had hitherto been unanticipated. This discovery underscores how the Kondo screening process can significantly alter *k*-space instabilities of Kondo lattice systems.

## Methods

### Film growth and characterizaion

Single crystalline, epitaxial, atomically smooth thin films of (001) YbAl_3_ and LuAl_3_ were synthesized on MgO substrates in a Veeco GEN10 MBE system with a liquid nitrogen cooled cryoshroud at a base pressure better than 2 × 10^−9^ Torr. Prior to growth, MgO substrates were annealed in vacuum for 20 min at 800 °C and a 1–2 nm thick aluminum (Al) buffer layer was deposited at 500 °C. Lu/Yb and Al were co-evaporated from Langmuir effusion cells at a rate of ≈0.4 nm/min onto a rotating substrate between 200 and 350 °C with real-time reflection high-energy electron diffraction (RHEED) monitoring. Due to the co-evaporation growth, the surface termination was not deliberately controlled. After growth, the films were immediately transferred under ultra-high vacuum to an ARPES chamber for measurements. All ARPES data presented in this study were performed on 30 nm thick LuAl_3_ thin films with a 1.2 nm thick Al buffer layer, or on 20 nm thick YbAl_3_ thin films with 20 nm thick LuAl_3_ and 1.2 nm thick Al buffer layers. The ARPES spectra did not show any thickness dependence for LuAl_3_/YbAl_3_ layers that were more than 10 nm thick, the minimum thickness for this study. For further details regarding film growth and characterization see ref. ^20^.

### In situ ARPES and XPS

After growth, thin film samples were immediately transferred within 5 min through ultra-high vacuum into an analysis chamber consisting of a VG Scienta R4000 electron analyzer, VUV5000 helium plasma discharge lamp and a dual anode x-ray source for ARPES and XPS measurements. The base pressure of the analysis chamber was better than 5 × 10^−11^ Torr. ARPES measurements were performed using He I*α* (*hν* = 21.2 eV) and He II*α* (*hν* = 40.8 eV) photons, while Al K*α* (*hν* = 1486.6 eV) photons were utilized for collecting XPS data. A polycrystalline gold reference, in electrical contact with the sample was used to determine position of the Fermi level and the energy resolution.

### DFT calculations

DFT calculations of the band structure and Fermi surface of LuAl_3_/YbAl_3_ with were performed using full potential linearized augmented plane wave method as implemented in the Wien2k software package^33^. The exchange and correlation effects were taken into account within the generalized gradient approximation^34^. Relativistic effects and spin-orbit coupling were included. For LuAl_3_, we found that an on-site Coulomb repulsion of *U* = 2.08 eV^35^ would give good agreement between the Lu 4*f* orbitals to the binding energies of the core levels measured in experiment. However, the value of *U* had no impact on the near-*E*_F_ electronic structure in LuAl_3_. For YbAl_3_, calculations were performed both with and without application of *U* to the Yb 4*f* orbitals, which was found to have a significant impact on the near-*E*_F_ electronic structure. (Supplementary Fig. 4).

### RXES

RXES spectra were collected at the Cornell High Energy Synchrotron Source (CHESS) at the C1 bend magnet beamline under ring conditions of 5.3 GeV and 100 mA. Incident x-ray radiation was monochromated using a Rh mirror and a sagittal focus double Si(2 2 0) crystal monochromator. The incident energy was calibrated using a Cu foil. The x-ray emission was monochromated and focused using five spherically bent Ge(6 2 0) crystals in the Rowland geometry by using the CHESS dual array valence emission spectrometer^36^. X-rays were finally collected with a Pilatus 100 K area detector (Dectris). Use of an area detector offered significant advantages for the current experiment in terms of ease of alignment and reliable background subtraction. Two regions of interest (ROIs) were chosen, one containing more than 95% of the emission signal and another centered on the first ROI, but four times in size. The larger ROI was used to correct for the average background counts as

1 $${I_{{\rm{corrected}}}} = {I_{{\rm{RO}}{{\rm{I}}_{\rm{1}}}}} - {\rm{Are}}{{\rm{a}}_{{\rm{RO}}{{\rm{I}}_{\rm{1}}}}} \times \left( {\frac{{{I_{{\rm{RO}}{{\rm{I}}_{\rm{2}}}}} - {I_{{\rm{RO}}{{\rm{I}}_{\rm{1}}}}}}}{{{\rm{Are}}{{\rm{a}}_{{\rm{RO}}{{\rm{I}}_{\rm{2}}}}} - {\rm{Are}}{{\rm{a}}_{{\rm{RO}}{{\rm{I}}_{\rm{1}}}}}}}} \right),$$

where ROI_1_ and ROI_2_ are the larger and smaller ROIs, respectively. *I*_ROI_ and Area_ROI_ denotes the intensity and area corresponding to the region of interest, respectively, while *I*_corrected_ is the corrected intensity after background subtraction.

Measured counts were further corrected for variations in incident photon flux by normalizing with the measured incident flux using a N_2_-filled ionization chamber placed upstream of the sample stage. X-ray emission energy was calibrated measuring the K_*α*1_ and K_*α*2_ lines of a Cu foil. The overall energy resolution of the setup was determined to be better than ≈3 eV measuring quasi-elastic scattering from a polyimide sample. To minimize photodamage, a fast shutter was placed upstream of the ionization chambers that would only open during active data taking, thus minimizing x-ray dosage of the samples. No photodamage was observed even after taking more than four scans (the maximum number of scans used for measurements at a particular spot) at a single spot. The sample was mounted on a closed cycle cryostat with base temperature of 45 K. A helium filled bag covering most of the x-ray path between the sample, analyzer, and detector was placed to reduce air attenuation along the x-ray path.

### Data availability

Data that support the findings of this study are available from the corresponding author upon request.

## Electronic supplementary material

Supplementary Information
