# Supplementary material for: Lifshitz transition from valence fluctuations in YbAl3
Source: Nat Commun. 2017 Oct 11;8:852. doi: 10.1038/s41467-017-00946-1 (PMC5636910; doi:10.1038/s41467-017-00946-1)
Supplement: Supplementary file 1 — Supplementary Information [file 41467_2017_946_MOESM1_ESM.docx]

**Supplementary Note 1: Determination of out-of-plane momentum (*k*_z_)**

Our ARPES measurements access a two-dimensional projection of the three-dimensional Brillouin zone centered around a *k_z_* value that depends on the incident photon energy.

$k_{z}= \sqrt{\frac{2m}{\hbar^{2}}\left( E_{h\nu}+V_{0}-\phi\right)-k_{||}^{2}}$ (1)

where, *E_hν_* is the incident photon energy, (*V_0_* - *ϕ*) is the inner potential, and *k_||_* is the in-plane momentum.

Both YbAl_3_ and LuAl_3_ have a three-dimensional cubic structure and therefore, are expected to have strong dispersion in their electronic band structure along *k_z_.* We estimate *k_z_* by comparing the measured band structure of LuAl_3_ with the results from density functional theory (DFT) calculations. Such an approach, however, poses a challenge for YbAl_3_ as it is well known that DFT+U methods perform poorly for Kondo lattice systems because they fail to satisfactorily account for the strong correlations of the partially filled *f* shell [1]. Nevertheless, such a method should perform reasonably well for LuAl_3_, which is a conventional metal system with a filled *f* shell.

In **Fig. 1a-c,** of the main text and in the **Supplementary Fig. 1** we show that the simulated two-dimensional Fermi surface topology at k_z_ = Γ and the corresponding band dispersion along (0, 0 - 0, π) are in good agreement with the measured Fermi surface and ARPES spectra, respectively, obtained using a photon energy of 21.2 eV. Furthermore, comparing our data against calculated Fermi surface contour plots and band dispersions for different *k_z_* values we establish that the best agreement is obtained for *k_z_* ~ Γ (**Supplementary Fig. 1b-d**). Similar exercise for data collected using 40.8 eV photons leads us to conclude that we are probing a two-dimensional momentum region centered at *k_z_* ~ X (**Supplementary Fig. 1b,e-f**).

A Fermi surface map taken with a photon energy of 40.8 eV in LuAl_3_ is shown in **Supplementary Fig. 1e** (right panel), where the electron pocket centered at (π, π) is closely reproduced by the calculation, with exception of the star-shaped hole like feature at X, which we ascribe to weak photoemission matrix elements. Nevertheless, comparing measured band dispersions along a momentum cut shown in **Supplementary Fig. 1b** (blue line) with our DFT calculations we also obtain the best agreement for k_z_ = X.

Similar band dispersions are observed in YbAl_3_ for broadly dispersive, primarily Al-derived bands at higher binding energies for identical momentum cuts and photon energies, as shown in **Fig. 1f** (main text), indicating we are probing identical *k_z_* region with a particular photon energy in both LuAl_3_ and YbAl_3_. This correspondence can be seen in many different *E* vs. *k* plots taken at different momentum regions of the Brillouin zone, shown in **Supplementary Fig. 3d-k**, assuming a shift in chemical potential due to different average rare-earth valence in these two systems. In fact, a similar two-dimensional electronic structure over the whole Brillouin zone is observed for LuAl_3_ at 0.95 eV binding energy and YbAl_3_ at 0.22 eV binding energy (**Supplementary Fig. 3a,b**).

As expected, DFT fails to reproduce the experimental low-energy electronic structure of YbAl_3_ (within ~ 1 eV), due to the hybridization of the light bands with the renormalized Yb 4*f* as shown in **Supplementary Fig. 2**. Nevertheless, the band dispersions at higher binding energies away from the 4*f* states also give the closest agreement with k_z_ = Γ and k_z_ = X with measurements using He Iα and He IIα photons, respectively, as also found in its uncorrelated analogue LuAl_3_. Therefore, the experimentally observed correspondence in the electronic structures of LuAl_3_ and YbAl_3_, along with a detailed comparison with our DFT calculations establishes that we are probing identical k_z_ values for both LuAl_3_ and YbAl_3_, which is k_z_ = Γ for He Iα (21.2 eV) and k_z_ = X for He IIα (40.8 eV) photon energies, respectively. Using equation 1, from the estimated *k_z_* values corresponding to the excitation energies of 21.2 eV and 40.8 eV, we obtain the inner potential of the (001) LuAl_3_ / YbAl_3_ surface, V_0_ - ϕ = 13.66 eV.

**Supplementary Note 2: Determination of Yb valence in YbAl_3_**

Temperature-dependent Yb valence in YbAl_3_ is determined by x-ray photoemission spectroscopy (XPS) and resonant x-ray emission spectroscopy (RXES).

In the case of a mixed-valence system, such as YbAl_3_, x-ray photoemission spectrum is expected to consist of two different final state multiplets corresponding to different initial valence configurations. For YbAl_3_, both 4*f*^13^ (peaks between 0 - 2 eV) and 4 *f*^12^ (peaks between 5 - 12 eV) final states are observed, separated by U*_ff_* ~ 6.5 eV, shown in **Supplementary Fig. 5a** that correspond to Yb^2+^ 4*f*^14^ and Yb^3+^ 4*f*^13^ initial states, respectively. The average Yb valence ν is determined by evaluating the integral spectral weight corresponding to 4*f*^13^ (I^2+^) and 4*f*^12^ (I^3+^) final states as [2,3]

$\nu= 3- \frac{\frac{I^{2+}}{14}}{\frac{I^{2+}}{14}+\frac{I^{3+}}{13}}$ (2)

In **Supplementary Fig. 5b**, we show the estimated variation in the average Yb valence in YbAl_3_ as a function of sample temperature. It should be noted that the measured photoemission intensity can be strongly influenced by matrix element and final state effects. Furthermore, to have a good estimation of the background intensity, knowledge of the non-4*f* spectral weight at that particular photon energy and contributions from inelastically scattered electrons is required, making the estimation process tricky. Indeed, there is a large distribution in the reported absolute Yb valence in YbAl_3_, dependent primarily on the nature of the probe and on the quality of the sample surface [2-7]. Nonetheless, a relative change in intensity as a function of temperature can be determined much more precisely leading to an accurate determination of temperature-dependent changes in the Yb valence, because a change in temperature is expected to negligibly affect the factors modulating measured photoemission intensity and non-4*f* spectral weight, which is shown in **Fig. 2c** of the main text.

RXES is a bulk-sensitive photon-in photon-out technique that can be used as an alternative to XPS for determination of rare-earth valence in Kondo lattice systems. The incident photon energy is tuned to the Yb LIII absorption edge, thus resonantly enhancing the signal from Yb in YbAl_3_, making it element specific. Ground states with Yb^2+^ and Yb^3+^ valence states undergo slightly different transitions resulting in the corresponding absorption edges being separated in energy by ~ 8 eV in YbAl_3_ [2,3,8]. Measurement of the absorption spectral weight corresponding to different Yb valence configurations leads to an estimate of the average Yb valence. In RXES, the emitted x-rays are energy resolved and only a particular decay channel is collected, resulting in greater resolving power compared to x-ray absorption spectroscopy (XAS) [2,3,9].

In **Supplementary Fig. 6c** we show normalized spectra taken at the L_α1_ emission line both at 300 K and 45 K. It is clearly seen that the contribution of the Yb^2+^ absorption peak increases at the lower temperature indicating that the average Yb valence decreases with temperature. To obtain a quantitative estimate of the average Yb valence, RXES spectra were fitted with Voigt functions for absorption peaks corresponding to Yb^2+^ and Yb^3+^. Absorption features corresponding to Yb^3+^ exhibit a double-peak structure requiring a two-component Voigt function. The double-peak structure for the Yb^3+^ component has been seen in other Yb intermetallic compounds such as YbCuAl [10] and Yb_2_Ni_12_P_7_ [11], and is ascribed to the crystal field splitting of the unoccupied Yb 5*d* states. A series of arctan-like functions capturing the edge jumps corresponding to the absorption edges is used to estimate the background. The average Yb valence is estimated using the following formula

$\nu= 2+ \frac{I(3^{+})}{I\left( 2^{+} \right)+I(3^{+})}$ (3)

where, I(2^+^) and I(3^+^) are respective intensities of Yb^2+^ and Yb^3+^ components [3,10], and is found to be 2.83 ± 0.01 at 300 K and 2.78 ± 0.01 at 45 K. Thus, from 300 K to 45 K the average Yb valence in YbAl_3_ decreases by ~ 0.05, consistent with results from similar measurements on YbAl_3_ single crystals [2,6,7].

**Supplementary Note 3: Fits to YbAl_3_ data**

Energy distribution curves (EDCs) at different temperatures, as shown in **Supplementary Fig. 7a**, are fitted with a functional form consisting of Lorentzians multiplied by the corresponding Fermi-Dirac distribution and convoluted with a Gaussian representing instrumental broadening, the full width at half maximum (FWHM) of which is estimated measuring a gold reference. A Shirley background that takes into account contribution from the inelastically scattered electrons is subtracted before the fitting process [12].

Similar fits have also been performed to extract crystalline electric field (CEF) split states for the EDCs shown in **Supplementary Fig. 8b,c**.

Momentum distribution curves (MDCs) at the Fermi energy, shown in **Supplementary Fig. 7b**, are fitted with a functional form consisting of two Lorentzians and a linear background. The MDC at EF taken at 21 K, in contrast to those taken at higher temperatures, exhibits only a single peak and hence, is fitted with a single Lorentzian. This further establishes that at 21 K the electron-like pocket centered at (0, 0) is completely lifted above the Fermi level and only the residual spectral weight is observed.

To access thermally occupied states above the Fermi level, the *E* vs. *k* plot obtained is divided by the corresponding resolution broadened Fermi-Dirac distribution, shown in **Supplementary Fig. 7c**, where the band bottom of the electron pocket is seen above the Fermi level.

**Supplementary Figure 1: Determination of *k_z_* in LuAl_3_. a,** DFT-calculated three-dimensional Fermi surface of LuAl_3_ with *U* = 0. Momentum regions centered at *k_z_* = Γ accessed by He Iα (21.2 eV) and at *k_z_* = X accessed by He IIα (40.8 eV) photon energies are shown by green and yellow regions, respectively. **b,** Simulated two-dimensional Fermi surface maps from DFT calculations with *k_z_* smearing taken over the thickness of the slabs shown in **a,** and overlaid with extracted *k_F_*s from experiment are shown for both He Iα and He IIα. Measured Fermi surface map and corresponding *k_F_*s along with calculated Fermi surface contour plots for different *k_z_* values for **c,** 21.2 eV and **e,** 40.8 eV photon energies. Fermi surface contour plots corresponding to *k_z_* = 0 and *k_z_* = π/c are shown in bold in **c,** and **e,** emphasizing good agreement with the extracted *k_F_*s using He Iα and He IIα photons, respectively. *E* vs. *k* dispersion, extracted dispersion and calculated dispersions for different *k_z_* values **d,** for He Iα, corresponding to the momentum line cuts shown in red in **b** **f,** for He IIα, corresponding to the momentum line cuts shown in blue in **b**. Extracted dispersion from the *E* vs. *k* plots in **d,** and **f,** are in good agreement with the calculated dispersions that are shown in bold for *k_z_* = 0 and *k_z_* = π/c, respectively. For clarity, dispersions for only those bands that form the Fermi surface are shown for *k_z_* values different from *k_z_* = 0 and *k_z_* = π/c in the left panels of **d,** and **f,** respectively; some of the bands that remain completely below *E_F_*, which can be seen in ARPES experiment, are omitted from the plotted bands on the left.

**Supplementary Figure 2: Calculated and measured electronic structure in YbAl_3_. a,** DFT-calculated three-dimensional Fermi surface of YbAl_3_ with *U* = 0. Momentum regions centered at *k_z_* = Γ accessed by He Iα (21.2 eV) and at *k_z_* = X accessed by He IIα (40.8 eV) photon energies are shown by green and yellow regions, respectively. **b,** Projected two-dimensional Fermi surface maps centered at *k_z_* = Γ (He Iα) and *k_z_* = X (He IIα) over the momentum regions shown in **a.** Fermi wave vectors (*k_F_*s) extracted from photoemission measurements using He Iα and He IIα are overlaid on top. Measured Fermi surface map and corresponding *k_F_*s along with calculated Fermi surface contour plots for different *k_z_* values for **c,** 21.2 eV and **e,** 40.8 eV photon energies. Fermi surface contour plots corresponding to *k_z_* = 0 and *k_z_* = π/c are shown in bold in **c,** and **e,** respectively. These all show generally poor agreement, due to the inadequate treatment of the strong correlations of the Yb 4*f* orbitals in the DFT calculation. *E* vs. *k* plot, extracted dispersion and calculated dispersions for different *k_z_* values **d,** for He Iα, corresponding to the momentum line cuts shown in red in **b f,** for He IIα, corresponding to the momentum line cuts shown in blue in **b.** For clarity, dispersions for only those bands that form the Fermi surface are shown for *k_z_* values different from *k_z_* = 0 and *k_z_* = π/c in the left panels of **d,** and **f,** respectively.

**Supplementary Figure 3: Similarity in electronic structure between LuAl_3_ and YbAl_3_.** Four-fold symmetrized two-dimensional electronic structure of **a,** LuAl_3_ at 0.95 eV binding energy and of **b,** YbAl_3_ at 0.22 eV binding energy, both obtained with a photon energy 21.2 eV (He Iα) **c,** The surface Brillouin zone of LuAl_3_ and YbAl_3_ showing high symmetry points and location of the ARPES cuts in momentum space. *E* vs. *k* plots corresponding to the ARPES cuts in panel **c** are shown in **d-g,** for LuAl_3_ and in **h-k,** for YbAl_3_. White dotted lines are guides to the eye highlighting the similarity in band dispersions in LuAl_3_ and YbAl_3_. White arrows in panels **d** and **h** indicate the binding energies at which two-dimensional maps shown in **a** and **b** are taken, respectively.

**Supplementary Figure 4:** **Evolution of the calculated electronic Fermi surface at Γ across LuAl_3_ and YbAl_3_.** Calculated three-dimensional Fermi surface for **a,** LuAl_3_ **b,** YbAl_3_ with U = 0 **c,** YbAl_3_ with U = 5.46 eV applied to Yb 4*f* orbitals. Corresponding two-dimensional Fermi surface contour plots at k_z_ = Γ for **d,** LuAl_3_ **e,** YbAl_3_ with U = 0 **f,** YbAl_3_ with U = 5.46 eV. Corresponding energy-momentum dispersion for a momentum cut (black line) shown in **d,** for **g,** LuAl_3_ **h,** YbAl_3_ with U = 0 **i,** YbAl_3_ with U = 5.46 eV. Dispersion of the electron pocket that forms the quasi-spherical Fermi surface centered at Γ is shown in red. It is clearly seen that the binding energy of the Yb 4*f* electronic states strongly influence the size of the quasi-spherical Fermi surface.

**Supplementary Figure 5:** **XPS spectra and Yb valence in YbAl_3_. a**, Evolution of XPS spectra with temperature. A Shirley-type background used to estimate contributions from inelastically scattered electrons is shown for the XPS spectra taken at 250 K (black line). **b,** Yb valence as a function of temperature evaluated estimating spectral intensity corresponding to 4*f*^13^ and 4*f*^12^ final states. Error bars include statistical error of one standard deviation from the fitting process and, also error in the estimated value due to variability in the fit results by holding one and/or more than one peak positions constant during the multi-peak fit.

**Supplementary Figure 6:** **Resonant x-ray emission spectroscopy (RXES) on YbAl_3_ thin films. a,** Two-dimensional intensity map of the photon yield around the Yb L_α1_ (7.416 KeV) emission energy plotted as a function of incident energy and energy transfer into the sample (*E_in_* – *E_out_*) The line cut shown in green corresponds to the emission energy L_α1_ **b,** Intensity variation of the emission spectra with excitation energy at Yb L_α1_ emission energy with the corresponding fit showing contributions from Yb^2+^ and Yb^3+^ components and respective arc-tan like contributions capturing the edge jumps. The absorption feature corresponding to Yb^3+^ has a double peak structure, which is ascribed to the crystal field splitting of the Yb 5*d* band. **c,** Identical cut as in panel **b** at two different temperatures. Spectra are normalized to their corresponding maximum in intensity to highlight the enhanced contribution from Yb^2+^ at the lower temperature. **d,** Estimated Yb valence as a function of temperature as obtained from RXES. Error bars include statistical error of one standard deviation from the fitting process and, also error in the estimated value due to variability in the fit results by holding one / two peak positions constant during the multi-peak fit.

**Supplementary Figure 7: Fits to YbAl_3_ ARPES data. a,** Energy distribution curves (EDCs) at different temperatures obtained after integrating over a momentum region shown as red in *Fig. 3d,* (main text) along with their corresponding fits (red solid line). Individual contributions to the fitted spectrum (blue and green solid lines) is shown for one such fit. A Shirley background (violet line) has been subtracted out prior to the fitting process that accounts for inelastically scattered electrons. **b,** Momentum distribution curves (MDCs) at *E_F_* taken at different temperatures along with their corresponding fits. **c,** High resolution, high-statistics *E* vs. *k* plot at 21 K obtained after dividing by the resolution broadened Fermi function. The parabolic band (black solid line), is a guide to the eye for the dispersion of the electron-like pocket at Γ.

**Supplementary Figure 8:** **Crystalline Electric Field (CEF) split states in YbAl_3_ a,** High-resolution *E* vs. *k* plot along (0, 0) - (0, π) in YbAl_3_ taken at 21 K showing dispersive CEF split states. **b,** Waterfall EDC plot with the extracted dispersion of the CEF split states overlaid on top. **c,** EDCs taken at the momentum region shown by red arrows in panel **a,** also highlighted in red in panel **b,** with their corresponding fits showing individual contributions. A Shirley-type background that has been subtracted prior to the fitting process to account for the inelastically scattered electrons is shown in brown. The binding energy separation between the CEF split states (Δ_1_, Δ_2_) at the two different *k* points are also indicated, highlighting their *k* dependence. In addition to statistical error of one standard deviation from the fitting process, error bars also include variability in the fit results by holding one and/or two peak positons constant in the multi-peak fitting process.

**Supplementary References**

[1] Zwicknagl, G. Quasiparticles in heavy fermion systems, *Physica Scripta*, **T49**, 34-41 (1993)

[2] Moreschini, L. *et al.,* Comparison of bulk-sensitive spectroscopic probes of Yb valence in Kondo systems, *Phys. Rev. B*, **75,** 035113 (2007)

[3] Kummer, K. *et al.,* Intermediate valence in Yb compounds probed by 4*f*  photoemission and

resonant inelastic x-ray scattering, Phys. Rev. B, **84,** 245114 (2011)

[4] Tjeng L. H. *et al.,* Temperature dependence of the Kondo Resonance in YbAl_3_, *Phys. Rev. Lett.,* **71,** 1419-1422 (1993)

[5] Suga, S. *et al.,* Kondo Lattice Effects of YbAl_3_ Suggested by Temperature Dependence of High-Accuracy High-Energy Photoelectron Spectroscopy, *J. Phys. Soc. Jpn.,* **74,** 2880-2884 (2005)

[6] Bauer, E. *et al.,* Anderson lattice behavior in Yb_1-x_Lu_x_Al_3_, *Phys. Rev. B*, **69,** 125102 (2004)

[7] Lawrence, J. M., Kwei, G. H., Canfield, P. C., DeWitt, J. G., Lawson, A. C. LIII x-ray absorption in Yb compounds: Temperature dependence of the valence, *Phys. Rev. B*, **49**, 1627-1631 (1994)

[8] Kumar, R. S. *et al.,* Pressure-induced valence change in YbAl_3_: A combined high-pressure inelastic x-ray scattering and theoretical investigation, *Phys. Rev. B*, **78,** 075117 (2008)

[9] Glatzel, P., and Bergmann, U. High resolution 1*s* core hole X-ray spectroscopy in 3*d* transition metal complexes: electronic and structural information, *Coord. Chem. Rev.,* **249**, 65-95 (2005)

[10] Yamaoka, H. *et al.,* Valence transitions in the heavy-fermion compound YbCuAl as a function of temperature and pressure *Phys. Rev. B*, **87,** 205120 (2013)

[11] Jiang, W. B. *et al.,* Crossover from a heavy fermion to intermediate valence state in non-centrosymmetric Yb_2_Ni_12_(P,As)_7_, *Sci. Rep.,* **5,** 17608 (2015)

[12] Shirley, D. A. High-Resolution X-Ray Photoemission Spectrum of the Valence Bands of Gold, *Phys. Rev. B*, **5,** 4709-4714 (1972)
